# Genomic Patterns are Associated with Different Sequelae of Patients with Long‐Term COVID‐19

**DOI:** 10.1002/advs.202407342

**Published:** 2024-12-31

**Authors:** Nan Zhang, Xizi Luo, Xiangwen Ji, Tian Tian, Runze Wu, Shishun Zhao, Guoqing Wang

**Affiliations:** ^1^ State Key Laboratory for Diagnosis and Treatment of Severe Zoonotic Infectious Diseases Key Laboratory of Pathobiology Ministry of Education China‐Japan Union Hospital of Jilin University Changchun 130033 China; ^2^ College of Mathematics Jilin University Changchun 130012 China; ^3^ State Key Laboratory for Zoonotic Diseases Key Laboratory for Zoonosis Research of the Ministry of Education College of Veterinary Medicine Jilin University Changchun 130062 China; ^4^ College of Basic Medical Sciences Jilin University Changchun 130021 China; ^5^ Department of Cardiology and Institute of Vascular Medicine Peking University Third Hospital 49 Huayuanbei Road Beijing 100191 China

**Keywords:** exon sequencing, genome mutation pattern, genomic fingerprinting framework, long‐term COVID‐19, non‐negative matrix factorization

## Abstract

In the post‐large era, various COVID‐19 sequelae are getting more and more attention to health problems. Although the mortality rate of the COVID‐19 infection is now declining, it is often accompanied by new clinical sequelae with different symptoms such as fatigue after infection, loss of smell. The degree of age, gender, virus infection seems to be weakly correlated with clinical symptoms. Human genetic variation plays a significant role in the sequelae of the COVID‐19 infection. This study aims to analyze the genomic differences between individuals with different COVID‐19 sequelae. In this study, the exomes of 97 patients with Omicron with 8 unique clinical manifestations are sequenced, and conducted a systematic analysis. Based on non‐negative matrix factorization algorithms, the trinucleotide mutation spectrum of four long‐term COVID‐19 genomes is summarized and found that individuals with different clinical symptoms have unique DNA mutation patterns and indel patterns. By constructing a Genomic Fingerprinting Framework, the driver genes of variation in each symptomatic population are deciphered and analyzed. This study showed that population‐specific mutational fingerprint differences are the main cause of heterogeneity in long‐term COVID‐19 sequelae. This study provides new ideas and insights into the causes of the long‐term COVID‐19 sequelae.

## Graphical abstract

This study sequenced and analyzed the exomes of long COVID‐19 patients exhibiting eight distinct clinical manifestations. The study found that individuals with different clinical symptoms has unique DNA mutation patterns. The findings suggest that the significant number of mutations in the host genetic background are the decisive factors leading to the post‐infection sequelae.

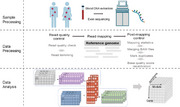

## 1 Introduction

The ongoing pandemic caused by severe acute respiratory syndrome coronavirus 2 (SARS‐CoV‐2), commonly known as COVID‐19, has become a serious global public health problem.^[^ ^1^ ^]^ Although the mortality rate after SARS‐CoV‐2 infection is declining, various persistent sequelae, such as fatigue, loss of smell and taste, and joint pain, continue to affect people's quality of life.^[^ ^2^ ^]^

The clinical symptoms of COVID‐19 vary among individuals. Age, sex, and virus variation appear to be the main reasons for differences in the long‐term sequelae of COVID‐19.^[^ ^3^ ^]^ For example, long‐term COVID‐19 sequelae in young people, such as headaches and loss of smell,^[^ ^4^ ^]^ were more significant. Among female patients, the COVID‐19 sequelae often manifest as brain fog.^[^ ^5^ ^]^ Omicron BA.5 is more likely to cause muscle soreness, whereas Omicron XBB.1.16 is more likely to cause diarrhea and conjunctivitis.^[^ ^6^ ^]^ However, explaining the diversity of complications in patients with long‐term COVID‐19 only through differences in viral strains, age, and sex is superficial and unsatisfactory. Individual differences in the host are the main factors affecting the diversity of long‐term COVID‐19 sequelae. Host genetic differences play a decisive role in the diversity of long‐term COVID‐19 sequelae. For example, Professor Janie discovered that the Rs7688383 mutation in *UGT2A1*/*UGT2A2* is closely related to loss of smell.^[^ ^7^ ^]^ *MUC5B* Rs35705950 is strongly associated with an increased risk of idiopathic pulmonary fibrosis.^[^ ^8^ ^]^ Currently, research methods on genetic variation in individuals with COVID‐19 are mainly based on genome‐wide association studies, Mendelian randomization, and META analysis to calculate the correlation between a single mutation site and symptom or severity. However, focusing only on the variation in a single mutation site or gene to explain the severity and symptoms of long‐term COVID‐19 is impractical.

**Figure 1:**
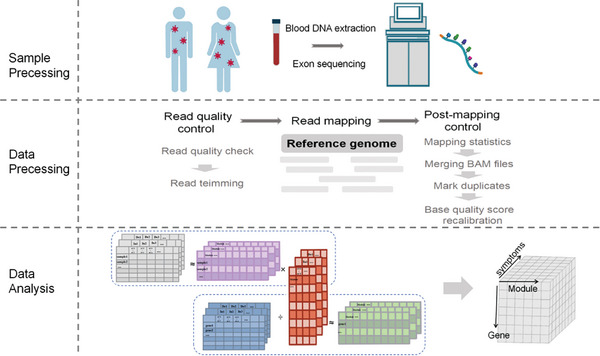
Workflow chart of GFPF model in this study.

Therefore, in our study, we analyzed the differences between different groups of people with post‐COVID‐19 syndrome dominated by Omicron by summarizing the mutation signature sets of each symptom in the host genome. We collected 97 patients with long‐term COVID‐19 (mainly Omicron infection) with 8 specific clinical manifestations for exome sequencing, summarized mutation information, drew 4 trinucleotide mutation maps related to long‐term COVID‐19, and found that each symptom group unique Mutation Patterns (CMPS). We constructed a genomic fingerprint identification framework, normalized the scoring of the symptom‐specific mutation pattern set and the indel feature set, deciphered the driver genes of each symptom, and then explained the reasons for the heterogeneity of COVID‐19 symptoms. The research framework of this study is shown in **Figure** **1**. Our study shows that unique mutation patterns in host genetic information are the main cause of heterogeneity in COVID‐19 symptoms. Our speculation analyzes the mechanism of COVID‐19 symptoms from the perspective of genetic mutation patterns.

## 2 Result

### 2.1 Heterogeneity of Long‐Term COVID‐19 Sequelae is Unrelated to Age, Sex, and Virus Subtype

We included the clinical information of 97 patients who tested positive for COVID‐19 using reverse transcription polymerase chain reaction at the Second Affiliated Hospital of Jilin University and Jilin University China‐Japan Joint Hospital in Changchun, China, between November 2022 and February 2023. Using viral gene sequence testing, the infected subtype was Omicron BA.5.2 or BF.7. We collected the symptoms and basic information of the patients after the infection, as shown in **Table** **1**. After the COVID‐19 infection, 59.8% of patients had loss of taste, 55.7% had brain fog, and 63.9% had muscle soreness. To determine whether external factors were the main cause of heterogeneity in the long‐term clinical manifestations of COVID‐19, we used chi‐square tests to assess the significance of sex, blood type, body mass index (BMI), and viral subtype on clinical characteristics. As shown in Table 1, correlation analysis between sex, age, BMI, viral infection subtype, and clinical manifestations was not significant. Therefore, we believe that external factors and viral subtypes are not the primary reasons for the heterogeneity in clinical symptoms after infection.

**Table 1:** Analysis of the correlation between the sequelae of long‐term COVID‐19 and patient gender, age, BMI, and virus subtype.

Index		Sex		Blood				BMI				Virus	
Clinic		Female	Male	A	AB	B	O	<18.5	18.5–24.9	24.9–27.9	≥27.9	BA.5.2	BF.7
Brain fog	Occur	27	27	14	10	13	17	15	7	16	16	24	30
	Not occur	21	22	12	8	9	14	9	10	18	6	24	19
	*p*‐value	1		0.9855				0.1377				0.3638	
Muscle aches	Occur	29	33	18	9	14	21	11	12	25	14	30	32
	Not occur	19	16	7	10	8	10	13	5	9	8	18	17
	*p*‐value	0.6177		0.2506				0.165				0.9392	
Taste loss	Occur	32	26	15	11	13	19	15	8	24	11	24	34
	Not occur	16	23	11	7	9	12	9	9	10	11	24	15
	*p*‐value	0.2463		0.9925				0.2903				0.08186	
Palpitations	Occur	34	32	20	12	15	19	18	9	23	16	32	34
	Not occur	14	17	6	6	7	12	6	8	11	6	16	15
	*p*‐value	0.7144		0.6574				0.4678				0.9445	
Viral diabetes	Occur	38	41	23	15	16	25	19	13	26	21	38	41
	Not occur	10	8	3	3	6	6	5	4	8	1	10	8
	*p*‐value	0.7568		0.5706				0.2869				0.7568	
Reproductive disorders	Occur	42	38	22	14	17	27	19	14	28	19	38	42
	Not occur	6	11	4	4	5	4	5	3	6	3	10	7
	*p*‐value	0.307		0.7465				0.9377				0.5613	
Diarrhea	Occur	41	40	24	15	15	27	23	13	27	18	39	42
	Not occur	7	9	2	3	7	4	1	4	7	4	9	7
	*p*‐value	0.8193		0.1384				0.2936				0.7499	
Smell loss	Occur	39	36	20	14	17	24	19	14	27	15	37	38
	Not occur	9	13	6	4	5	7	5	3	7	7	11	11
	*p*‐value	0.5013		0.9999				0.6998				1	

### 2.2 Somatic Mutation Landscape of Patients with Long‐Term COVID‐19

**Figure 2:**
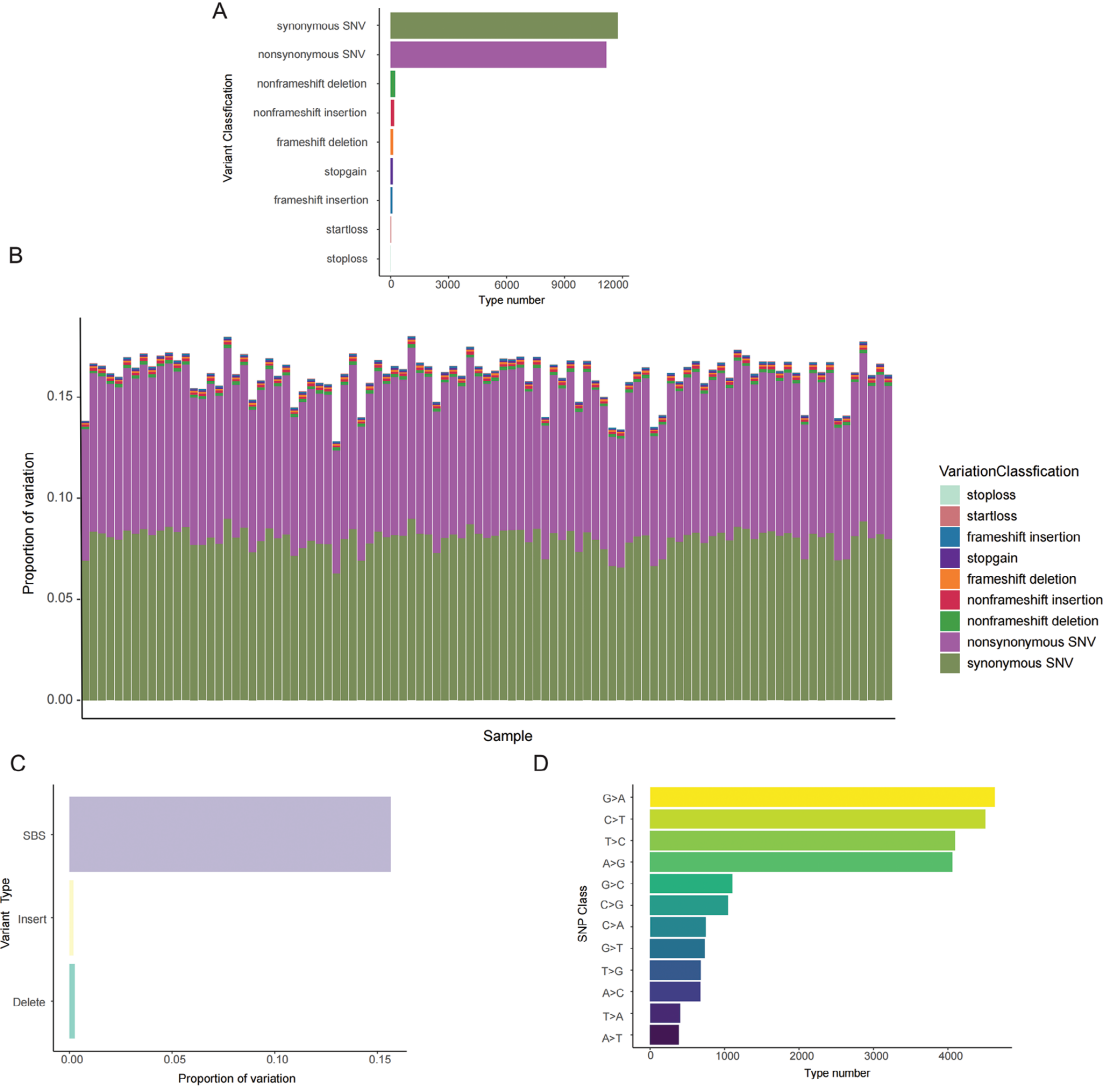
Summary of somatic mutations in long‐term COVID‐19 patients. A) Statistics of the number of mutations of each mutation type in patients with long‐term COVID‐19. B) Proportion of each mutation type in the samples of 97 long‐term COVID‐19 patients. C) Statistics on the proportion of single base mutations and insertion and deletions in patients with long‐term COVID‐19. D) Visualization of the top 12 single‐base mutation types in COVID‐19 patients.

To explore whether host genetic factors are the main factors causing clinical manifestations after COVID‐19, we performed whole‐exome sequencing (WES) of peripheral blood samples from 97 patients with long‐term COVID‐19. All samples were sequenced using a whole‐exome capture system covering 64 MB of the human genome. The sequencing depth of the sample was 100×, and the mapping rate was 99.1%. As shown in **Figure** **2**A,B, 11 754 somatic exon mutations are observed in the sample, including syno SNV11754 and non‐cyno SNV11172. We observed 270 insertions, of which 97 were frameshifts, and 173 were non‐frameshifts.^[^ ^9^ ^]^ We identified 353 deletions, 125 frameshift deletions, 228 non‐frameshift insertions, 14 stop, 28 start, and 113 stop mutations. As shown in Figure 2C, compared to insertion and deletion mutations, single‐base substitutions were the main mutation forms in the genetic variation. As shown in Figure 2D, among the 12 mutation types included in SNPS, the main single‐base mutation types are G>A, C>T, T>C, and A>G.

### 2.3 Mapping of Susceptibility Trinucleotide Mutation Patterns in Patients with Long‐Term COVID‐19

**Figure 3:**
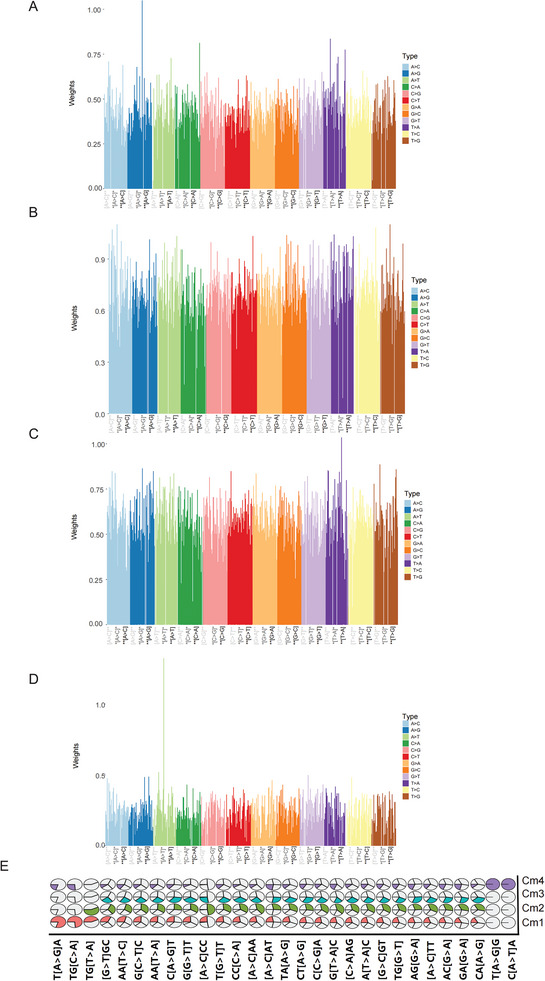
CMP deciphered from somatic cell genomes of long‐term COVID‐19 patients. A–D) CMP extraction from somatic cell genomes of long‐term COVID‐19 patients. Profiles for each CMP are displayed in the order of 12 alternative subtypes: A>C, A>G, A>T, C>A, C>G, C>T, G>A, G>C, G>T, T>A, T>C and T>G. The characters in the bottom tab represent the reference trinucleotide, colored according to the trinucleotide codon's preceding position, center position, and last position of the substituted nucleotide at the center position. E) Distribution of known major single‐base mutations in COVID‐19 in cmp.

A genome mutation pattern is a pan‐genome enrichment of repeated substitutions in the host gene sequence environment. A genome mutation pattern is hidden by the host in a high‐dimensional genome trinucleotide sequence and effectively reflects the rules of the host gene.^[^ ^10^ ^]^ To more accurately explore host genome mutation patterns associated with long‐term COVID‐19 susceptibility, we aimed to extract interpretable genomic trinucleotide mutation signatures from high‐dimensional data. Since the non‐negative matrix factorization algorithm ensures that the resulting coefficient matrix is non‐negative and unique, we apply the non‐negative matrix factorization (NMF) algorithm for dimensionality reduction analysis. As shown in **Figure** **3**A–D, we obtained four long‐term COVID‐19 infection‐related host genome patterns (Cmps) and their weight factor contribution systems (Table S1, Supporting Information). The trinucleotide mutation patterns associated with susceptibility to long‐term COVID‐19 were mainly T[A>G]A, TG[C>A], TG[T>A], [A>C]TT, TG[G>T], TA [A>G], and C[A>T]A. To verify the accuracy of Cmps, we screened 23andMe, COVID‐19 Host Genetics Initiative, Ancestry DNA Globally Harmonized System of Classification and Labelling of Chemicals, Penn Medicine Biobank, UK Biobank, and GeneOmicc databases, and 361 published articles related to COVID‐19 host genetic variation. We collected all available genetic variation information related to COVID‐19 and performed a cross‐analysis between the main mutation patterns of Cmps and existing COVID‐19 variants. As shown in Figure 3E, known COVID‐19 mutations coincided with Cmps and were distributed as CMP1–CMP4. Cmps accurately represent the genetic characteristics of COVID‐19‐related hosts.

### 2.4 Genetic Variations in Clinical Features of Patients with Long‐Term COVID‐19 are Specific

**Figure 4:**
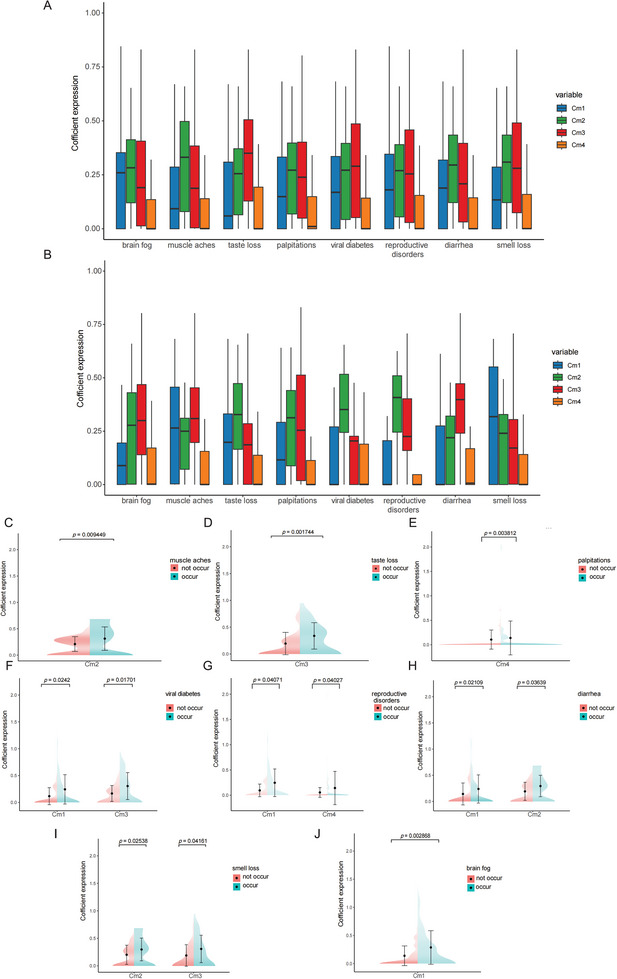
Analysis on the correlation between sequelae of COVID‐19 and CMPs. A,B) The differential contribution of CMPS in the germline genomes of samples with and without clinical sequelae. C–J) Demonstration of the correlation between clinical sequelae and significant contributions of CMPS in patients with long‐term COVID‐19 (The number of samples without this symptom in (C) is 35, the number of samples with this symptom in (C) is 62. The number of samples without this symptom in (D) is 39, the number of samples with this symptom in (D) is 58. The number of samples without this symptom in (E) is 31, the number of samples with this symptom in (E) is 66. The number of samples without this symptom in (F) is 18, the number of samples with this symptom in (E) is 79. The number of samples without this symptom in (G) is 17, the number of samples with this symptom in (G) is 80. The number of samples without this symptom in (H) is 16, the number of samples with this symptom in (H) is 81. The number of samples without this symptom in (I) is 22, the number of samples with this symptom in (I) is 75. The number of samples without this symptom in (J) is 43, the number of samples with this symptom in (J) is 54.

To explore the genome mutation pattern of each clinical symptom after COVID‐19 infection, we extracted the genome mutation pattern of each symptom and performed a correlation analysis with Cmps. As shown in **Figure** **4**A, the correlation between various symptoms and Cmps after a long‐term COVID‐19 infection was different. Therefore, individuals with various symptoms of long‐term COVID‐19 have unique DNA mutation fingerprints. As shown in Figure 4B, the main mutation patterns in individuals with loss of smell were related to Cm2 and Cm3, which were [A>C]TT and C[A>T]A, respectively. The main mutation pattern in individuals with loss of taste was related to Cm3, including AG[T>A] and [T>G]TC. The main mutation pattern in individuals with brain fog failure was related to Cm1: T[A>G]A and A[T>A]A. The main mutation patterns in individuals with muscle soreness and failure were related to Cm2, which were [A>C]TT and C[T>G]A. The main mutation pattern in individuals with palpitations was related to Cm4, which was C[A>T]A and [A>T]GG. The main mutation patterns in individuals with diarrhea were related to Cm1 and Cm2, which were T[A>G]A and [A>C]TT, respectively. The main mutation patterns in people with elevated viral blood sugar were related to Cm1 and Cm3, which are T[A>G]A and AG[T>A]. The main mutation patterns in individuals with reproductive disorders after viral infection were related to Cm1 and Cm4 and are T[A>G]A and C[A>T]A, respectively (Figure 4C–J).

**Figure 5:**
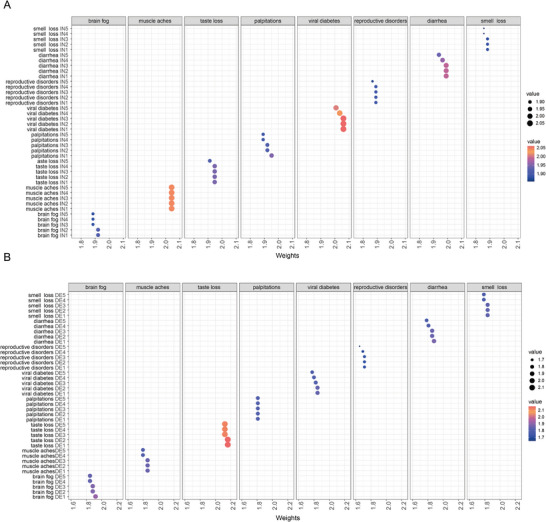
Extraction of indel features of various clinical sequelae in patients with COVID‐19. A) Visualization of insert module scores for each clinical sequelae of COVID‐19 patients. B) Visualization of delete module scores for each clinical sequelae of COVID‐19 patients.

In addition to focusing on the mutation patterns of various COVID‐19 symptoms, we extracted the insertion and deletion mutation patterns of each symptom group. As shown in **Figure** **5**A, people with loss of smell primarily had GTGTGC‐clustered base insertions. Individuals with brain fog primarily had CCGCAC main cluster‐based insertions. People with muscle soreness and loss of taste mainly had pyrimidine‐based irregular long‐cluster mutations (>10 bp). People with palpitations mainly had GTGTGC main cluster‐based insertions. People with viral hyperglycemia mainly had long mutations (>10 bp) starting with *AAG* or *GAG*. People with reproductive disorders after viral infections mainly had long mutations (>5 bp) starting from *AAG*/*GCG*. People with diarrhea mainly had long mutations (>5 bp) starting with *AAG*/*CGG*.

As shown in Figure 5B, ATTA‐ and CAGCACG‐based multi‐base cluster deletions were the main deletion patterns in individuals with smell loss, reproductive disorders, diarrhea, and heart palpitations. In addition, individuals with loss of smell and reproductive disorders also had GGTGTG polybase cluster deletions. Individuals with diarrhea and palpitations also had clustered deletions of multiple bases at the beginning of the GCT. Individuals with brain fog mainly have clustered deletions of multiple bases starting from TTG. GGTGTG and ATTA were the main polybase cluster deletions in individuals with muscle soreness. People with taste loss had GCGGCG and GGTGTG mutations, mainly multibase cluster deletions. The main deletion in individuals with elevated viral blood sugar levels after viral infection was ATTA, and GGTGTG was the main multi‐base cluster mutation. People with reproductive disorders after viral infection mainly lack ATTA, and CAGCACG has multi‐base cluster mutations.

### 2.5 Interpretation of Genome Mutation Characteristics of People with COVID‐19 Based on Mutational Contribution

**Figure 6:**
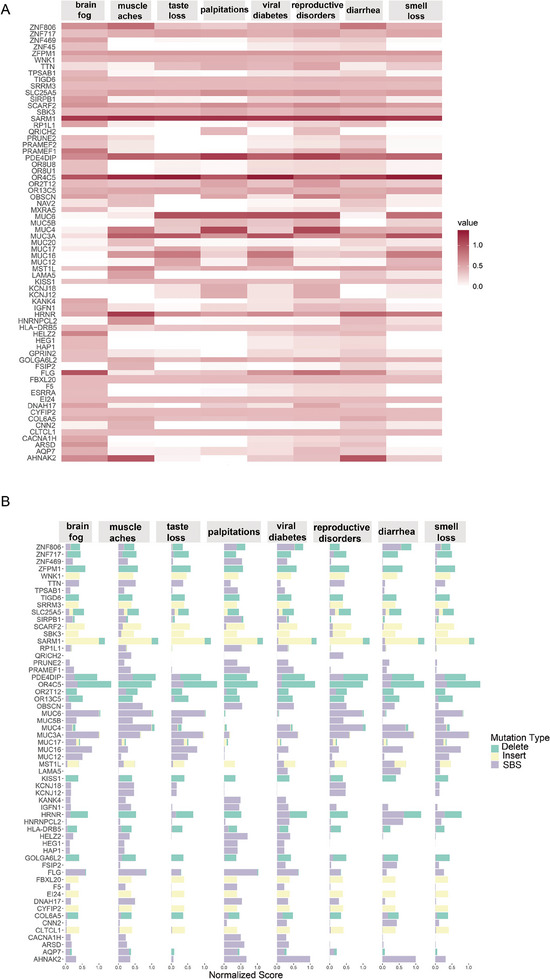
Based on the GSPF model, decipher the main contributing genes to the sequelae of long‐term COVID‐19. A) Based on the GFPF model, Calculation of heat map of major contributory genes in variation of various sequelae of long‐term COVID‐19. B) Details of the contribution of indels and base substitutions in the main mutated genes with the sequelae of long‐term COVID‐19.

To decipher the unique genomic variation of each COVID‐19 symptom in the host, we established a Genomic Fingerprinting Framework (GFPF). We supplemented the genetic variation information into genes and calculated the mutation, insertion, and deletion pattern scores of each gene according to the genetic variation weight using the NMF backward algorithm. We quantified each mutation score in the same dimension and performed standardization and cumulative summation. Based on these calculations, we obtained the main mutation driver gene set for each symptom, as shown in **Figure** **6**A,B. Based on the discovery of mutation contribution scores among people with taste loss, the mucin‐coding genes *MUC6*, *MUC3A*, *MUC16*, *MUC17*, and *MUC12* had the highest scores.^[^ ^11^ ^]^ The function of salivary mucin is imperfect, and taste sensitivity is reduced after viral stimulation. Among individuals with smell loss, olfactory receptor‐coding gene mutations, mainly *OR4C5*, *OR13C5*, *OR2T12*, and *OR4C3*, contributed the most, suggesting that many olfactory receptor dysfunctions occur. After viral infection, the host's olfactory function cannot be restored in time after the damage and the sensitivity and specificity of the olfactory senses are reduced.^[^ ^12^ ^]^ In patients with palpitations after COVID‐19, genes involved in cardiac contraction and relaxation, such as *OBSCN*, and *PDE4DIP*, had higher scores, suggesting a stable imbalance of CAMP/calcium in myocardial cells. During viral infection, the systolic function of the heart is damaged and cannot be repaired in time, causing palpitations in patients.^[^ ^13^ ^]^ Among individuals with brain fog, *FLG*, *HAP1*, and other cranial nerve repair gene mutation scores were higher. This causes the nervous system to be unable to repair quickly after the virus damages the brain tissue, leading to long‐term brain fog symptoms.^[^ ^14^ ^]^ In patients with diarrhea, a large number of mutations were observed in *MST1L* and *CYFIP2* intestinal barrier‐related genes.^[^ ^15^ ^]^ The virus invades the intestinal wall and increases intestinal permeability, thereby causing enteritis. In men with viral reproductive disorders, *DNAH17*, *WNK1*, testosterone secretion‐related gene *EI24*, and *QRICH2* spermatogenesis gene mutations have been reported.^[^ ^16^ ^]^ This suggests that this group of people is at risk of reproductive disorders. After the virus invades the reproductive organs, the testicles are damaged, accelerating the appearance of symptoms of reproductive disorders.^[^ ^17^ ^]^ In patients with COVID‐19‐related hyperglycemia, the mutation contribution score of a key regulator of pancreatic beta cell differentiation was higher.

## 3 Discussion

The heterogeneity of COVID‐19 sequelae has always been an issue of interest. In previous studies, the large differences in clinical manifestations after COVID‐19 infection were owing to different virus subtypes, patient sex, and age.^[^ ^18^ ^]^ However, in this study, we found that the virus strain, sex, age, blood type, and BMI were not significantly related to the long‐term COVID‐19 sequelae in patients. The unique genetic characteristics of the population are the main reasons for the different symptoms of COVID‐19.

In previous studies on genetic trait analysis, there has been a greater focus on point mutations, insertions, deletions, and other types of genetic variations. In this study, we focus on the frequency of genomic mutations, specifically examining the patterns of abundant and recurrent mutations, insertions, and deletions within the genome. Compared to single‐gene point mutations (single nucleotide polymorphisms [SNPs]), insertions, and deletions, mutation patterns can more effectively reflect the overall pattern lineage of genomic mutations, providing a more specific representation of the molecular perturbation characteristics of the host genome and offering a more comprehensive analysis of genomic variation patterns. Therefore, this study employed Non‐negative Matrix Factorization (NMF) to analyze the exome sequencing data of 97 long‐term COVID‐19 patients exhibiting symptoms such as olfactory loss, taste loss, palpitations, and diarrhea, thereby investigating the overall mutation landscape of long‐term COVID‐19 patients. By detecting and analyzing the exome sequencing data of 97 patients with long‐term COVID‐19 who suffered from smell loss, taste loss, palpitations, diarrhea, and other symptoms, we analyzed the overall mutation landscape of patients with long‐term COVID‐19. To observe the genetic variation patterns of various symptoms of COVID‐19, we extracted the mutation patterns of patients with each symptom and performed a correlation analysis with Cmps. We found that each symptomatic population had a unique fingerprint of genetic variation. For example, the main mutation pattern of anosmia population is related to Cm2 and Cm3, and there are GTGTGC cluster base insertion and ATTA, CAGCACG, GGTGTG multi‐base cluster deletion. The main mutation patterns of individuals with taste loss were related to Cm3, mainly pyrimidine irregular cluster mutation (>10 bp), GCGGCG, and GGTGTG multi‐base cluster deletion.

To decipher the genetic variation information for each symptom group, we established the GFPF algorithm to calculate the mutation burden score caused by each mutation pattern. We accurately assigned the mutation, insertion, and deletion patterns of each symptom group to genes and then attributed the genetic variation at the gene level. We attempted to determine the impact of each mutation type on the genetic variation. We found that compared with mutation patterns, the number of insertion and deletion patterns was smaller, and the scores were lower. To distribute insertions and deletions evenly, we adopted same‐dimensional processing, standardized the contribution coefficient of each pattern, summed each mutation pattern, and obtained the main driving variant genes in each symptom group. In individuals with olfactory loss, the olfactory receptor coding genes *OR4C5, OR13C5, OR2T12*, and *OR4C3* exhibited high mutation scores.^[^ ^19^ ^]^ This indicates that the olfactory receptor coding genes have undergone numerous mutations, leading to a deficiency in the olfactory function of the organism itself. The invasion of the virus further disrupts the olfactory system, resulting in patients persistently exhibiting symptoms of olfactory dysfunction. In patients with palpitations following SARS‐CoV‐2, the genes *OBSCN* and *PDE4DIP*, which are involved in cardiac contraction and relaxation, exhibited higher scores.^[^ ^20^ ^]^ Mutations in the *OBSCN* and *PDE4DIP* genes can lead to instability in calcium ion regulation during myocardial contraction and relaxation. This indicates that the host's cardiac contraction and relaxation functions are inherently impaired, and the invasion of the virus further damages cardiac tissue, exacerbating the dysfunction of these processes. This is a primary reason for the persistent palpitations experienced by patients. Among individuals with brain fog, cranial nerve repair gene mutation scores were higher.^[^ ^14^ ^]^ In patients with diarrhea, a large number of mutations were observed in intestinal barrier‐related genes.^[^ ^21^ ^]^ In men with viral reproductive disorders,^[^ ^22^ ^]^ testosterone secretion‐related gene and spermatogenesis gene mutations have been reported.^[^ ^23^ ^]^ In this study, we propose that SARS‐CoV‐2 infection merely exacerbates clinical symptoms, while the presence of numerous significant mutations within the host's olfactory, neurological, and other systems is the primary reason for the occurrence of post‐infection sequelae due to inherent dysfunction of these systems. The GFPF algorithm we proposed analyzes the formation mechanism of different long‐term COVID‐19 sequelae from the perspective of genetic mutation patterns.

In summary, the efficient GFPF algorithm we proposed not only provides an effective method for analyzing the intrinsic molecular mechanisms of various COVID‐19 sequelae but also provides new methods and ideas for exploring somatic mutation driver gene screening and genome‐based disease analysis. This study suggests that the unique DNA mutation fingerprint of each symptom group is the primary reason for the differences in the long‐term COVID‐19 sequelae. This study provides new insights and ideas for analyzing the heterogeneity of the clinical symptoms of COVID‐19 from the perspective of the genetics of hosts with COVID‐19.

## 4 Experimental Section

### Ethics Statement

This study was conducted in strict accordance with the principles of the Declaration of Helsinki. Medical records of patients previously diagnosed with COVID‐19 during clinical treatment and residual specimens obtained after clinical examinations were utilized for sequencing research. The study received approval from the Institutional Review Board of China‐Japan Union Hospital of Jilin University (Ethics Approval No.2024062014). All experiments were performed in compliance with the approved ethical and biosafety protocols. The detailed clinical information of the patients is shown in Table S2 (Supporting Information).

### Patient Recruitment

The patients who participated in this study were diagnosed with COVID‐19 between November 2022 and February 2023 and developed COVID‐19 sequelae within 2 months after infection. Written informed consent was obtained from all participants. Standard protocols were followed to extract genomic DNA from peripheral blood for genetic analysis.

### WES and Sequencing Data Preprocessing

Agilent's liquid‐phase chip capture system was used to efficiently enrich DNA in the human full exon region. After merging, each captured library was sequenced on an Illumina NovaSeq 6000 system with paired‐end 150 bp reads.

Quality control was performed on the fastq files of Omicron‐infected human genome data obtained using 97 WES, and the data was filtered and quality‐controlled using the fastp software. The BWA‐MEM algorithm was used for comparison with the reference genome and converted the data format using SAMtools. GATK was used for genetic analysis, screened for SNPs, and inserted and deleted insertions and deletions in the sequencing data by removing duplicate markers and recalibrating base quality scores. For the SNP sequencing data, the single‐base substitution position and specific three‐base type bound by the two bases before and after the experiment were considered. A sequencing dataset, D_576 × 97_ was formed for specific samples and single‐base substitutions based on 12 single‐base substitution types: the two bases before and after (4 × 4), substitution position (3), and number of samples (97). A sequencing dataset of genes and single‐base substitutionsG_gene × 576_ was constructed. For insert and deletion sequencing data, sample and insertion or deletion datasets and gene and insertion or deletion datasets were extracted and constructed based on the results of significant symptoms.

### WES and Sequencing Data Preprocessing—Model Decomposition and Reconstruction

GFPF algorithm:

Step 1: Calculate the weight coefficient

Step 2: Calculate the weight of the gene

For the sample and single‐base substitution datasets, the forward NMF algorithm was used to decompose, and the decomposition form is:

(1) $$D_{576\;\times \;97}=W_{576\;\times \;k}\;\times \;H_{k\;\times \;97}$$

H represents the number of samples and modules after dimensionality reduction, and W represents the coefficient proportion of a single base substitution in the module. k represents the choice of the number of modules in the NMF dimensionality reduction process. The selection of k was primarily determined based on the contour coefficient. The matrices W and H were obtained by decomposing matrix D using the `nmf` function from the NMF package in R. The magnitude of the weight coefficients in matrix W reflects the proportion of the corresponding mutation frequency represented by each module. In matrix H, the magnitude of the sample‐related coefficients indicates the proportion of each module within the sample. Inverse non‐negative matrix factorization was performed by combining specific salient symptoms, genes, and single‐base substitution datasets with the W matrix. The decomposition form is as follows:

(2) $$S_{\mathrm{gene}\;\times \;1}\approx G_{\mathrm{gene}\;\times \;576}/W_{1\;\times \;576}$$

S_G_ represents the mutation score of a gene. Therefore, based on the significant symptoms and specific single‐base substitution types, the mutation weights of the genes were extracted. Using a non‐negative matrix algorithm ensures the non‐negativity of the decomposition matrix during the dimensionality reduction process. Similarly, the same processing was performed on the insert and delete datasets.

### WES and Sequencing Data Preprocessing—Gene Scoring Algorithm

To better connect mutations with genes, the gene scores under single‐base substitution, insert, and delete were integrated. Through maximum value normalization and integration, the gene scores were calculated to better explain the connections between genes and symptoms. The specific form of optimal normalization is

(3) $$S_{\mathrm{sbs}}=\frac{x-\min\left(x\right)}{\max\left(x\right)-\min\left(x\right)}$$

(4) $$G_{\mathrm{score}}=\sum_{i=1}^{n}S_{i}/n$$

n represents the mutation type.

### Statistical Analysis

In clinical information research, to determine the association between sex, age, and other clinical information, a contingency table test was performed by using the chi‐square test function. To determine the correlation between clinical sequelae and the mutation module, the Wilcoxon test was used to determine whether a significant difference was observed between the symptoms and the mutation module. Statistical significance was set at *p* < 0.05. Wilcox.test and chisq.test were used for data statistical analysis in R 4.3.2.

## Conflict of Interest

The authors declare no conflict of interest.

## Author Contributions

N.Z. and X.L. contributed equally and are joint first authors of this work. G.W. design of research. X.J., T.T., R.W., and S.Z. analyzed the data. X.L. and N.Z. wrote the manuscript.

## Supporting information



Supporting Information

Supporting Information

## Data Availability

The data that support the findings of this study are available from the corresponding author upon reasonable request.
