# Supplementary material for: Genomic Patterns are Associated with Different Sequelae of Patients with Long‐Term COVID‐19
Source: Adv Sci (Weinh). 2024 Dec 31;12(8):2407342. doi: 10.1002/advs.202407342 (PMC11848565; doi:10.1002/advs.202407342)
Supplement: Supplementary file 1 — Supporting Information [file ADVS-12-2407342-s002.docx]

Supplementary Table 1 Genome mutation pattern and weight of COVID-19

| mutation type | CMP1  weight | CMP2  weight | CMP3  weight | CMP4  weight |
| --- | --- | --- | --- | --- |
| [A>C]AA | 0.527279023 | 0.987942557 | 0.622422318 | 0.244816429 |
| [A>C]CA | 0.366336638 | 0.898077873 | 0.761215005 | 0.11908773 |
| [A>C]GA | 0.70991596 | 0.647600468 | 0.656867144 | 0.383040104 |
| [A>C]TA | 1.08E-13 | 0.864835007 | 0.530537717 | 0.223899489 |
| [A>G]AA | 0.311669422 | 0.684540294 | 0.688520213 | 0.174315207 |
| [A>G]CA | 0.418812632 | 0.662972719 | 0.524326434 | 0.234100397 |
| [A>G]GA | 0.423169738 | 0.621908733 | 0.710844229 | 2.22E-16 |
| [A>G]TA | 0.471760101 | 0.535509841 | 0.695289311 | 0.078007209 |
| [A>T]AA | 2.22E-16 | 2.22E-16 | 2.22E-16 | 0.188447323 |
| [A>T]CA | 0.351633498 | 0.709761964 | 0.472851485 | 0.067592561 |
| [A>T]GA | 0.428430729 | 0.73089474 | 0.614810787 | 0.370398516 |
| [A>T]TA | 0.440907768 | 0.701912264 | 0.611807329 | 0.256366465 |
| [C>A]AA | 0.576750193 | 0.639406686 | 0.703940751 | 0.115240845 |
| [C>A]CA | 0.16622489 | 0.713834184 | 0.563449034 | 0.12912467 |
| [C>A]GA | 0.367298407 | 0.577097351 | 0.461927657 | 0.118454092 |
| [C>A]TA | 0.355848836 | 0.657388678 | 0.692717909 | 0.183686809 |
| [C>G]AA | 0.417735229 | 0.670402033 | 0.515953609 | 0.346002166 |
| [C>G]CA | 0.340856302 | 0.621545776 | 0.659707749 | 0.117118303 |
| [C>G]GA | 0.62604157 | 0.997942457 | 0.674309222 | 2.22E-16 |
| [C>G]TA | 0.648728186 | 0.75277745 | 0.815162096 | 2.22E-16 |
| [C>T]AA | 0.400457755 | 0.82239943 | 0.494393784 | 0.162705657 |
| [C>T]CA | 0.406764764 | 0.63573612 | 0.699423123 | 0.202182534 |
| [C>T]GA | 0.406959123 | 0.743638233 | 0.540123943 | 0.256855222 |
| [C>T]TA | 0.278534541 | 0.757177698 | 0.525374173 | 0.355687848 |
| [G>A]AA | 0.402773956 | 0.776939806 | 0.602539177 | 0.302070758 |
| [G>A]CA | 0.268386111 | 0.779831861 | 0.550951678 | 0.220674598 |
| [G>A]GA | 0.544375328 | 0.931136234 | 0.664823502 | 0.055309084 |
| [G>A]TA | 0.518714645 | 0.623409367 | 0.510168486 | 0.260696583 |
| [G>C]AA | 0.242606185 | 0.519644263 | 0.487726105 | 0.041379352 |
| [G>C]CA | 0.506232374 | 0.656650373 | 0.745157289 | 0.065667831 |
| [G>C]GA | 0.463263844 | 1.038833865 | 0.685654307 | 0.298038273 |
| [G>C]TA | 0.259186864 | 0.61958606 | 0.406462421 | 0.290736789 |
| [G>T]AA | 0.615128364 | 0.546581844 | 0.515609879 | 0.20465434 |
| [G>T]CA | 0.337942659 | 0.674556721 | 0.501568169 | 0.163561424 |
| [G>T]GA | 2.22E-16 | 0.199704384 | 2.22E-16 | 2.22E-16 |
| [G>T]TA | 2.22E-16 | 1.010164029 | 0.674908974 | 0.31353972 |
| [T>A]CA | 0.453416823 | 0.837755993 | 0.506856697 | 0.228572373 |
| [T>A]TA | 0.559697539 | 0.885782264 | 0.534249739 | 0.308844544 |
| [T>C]AA | 2.22E-16 | 2.22E-16 | 0.0981318 | 2.22E-16 |
| [T>C]CA | 0.414660687 | 0.512293253 | 0.623650531 | 0.148317406 |
| [T>C]GA | 0.514771001 | 2.22E-16 | 0.09670383 | 2.22E-16 |
| [T>C]TA | 0.340655002 | 0.738351233 | 0.459619487 | 0.208166998 |
| [T>G]AA | 2.22E-16 | 2.22E-16 | 2.22E-16 | 0.080763139 |
| [T>G]CA | 0.426175458 | 0.586895568 | 0.516681492 | 0.359141991 |
| [T>G]TA | 0.404586217 | 0.674108011 | 0.550138282 | 0.353059566 |
| A[A>C]A | 0.379798004 | 0.430190096 | 0.64258794 | 0.241343333 |
| A[A>G]A | 0.469975402 | 0.785843896 | 0.520976742 | 0.105319807 |
| A[A>T]A | 0.450751206 | 0.640248615 | 0.763033344 | 0.242333671 |
| A[C>A]A | 0.413993422 | 0.525189628 | 0.591615522 | 0.187348921 |
| A[C>G]A | 0.46053342 | 0.733194862 | 0.416136462 | 0.19667719 |
| A[C>T]A | 0.440223553 | 0.756679464 | 0.643350729 | 0.32717143 |
| A[G>A]A | 0.495784898 | 0.622860032 | 0.680535018 | 0.20407022 |
| A[G>C]A | 0.459233174 | 1.004355148 | 0.600329393 | 0.3023307 |
| A[G>T]A | 0.53018942 | 0.695630851 | 0.396413277 | 0.499170968 |
| A[T>A]A | 0.836816201 | 2.22E-16 | 2.22E-16 | 0.21444425 |
| A[T>C]A | 0.499480011 | 0.787723706 | 0.681613403 | 0.194950527 |
| A[T>G]A | 0.451098545 | 0.767750517 | 0.633710444 | 0.348619398 |
| C[A>C]A | 0.335035536 | 0.3882534 | 0.324992405 | 0.157374442 |
| C[A>G]A | 0.427955434 | 0.67300704 | 0.523331628 | 0.188513244 |
| C[A>T]A | 2.22E-16 | 2.22E-16 | 2.22E-16 | 1.324515472 |
| C[C>A]A | 0.489900434 | 0.583753867 | 0.527694399 | 0.431496626 |
| C[C>G]A | 0.548837946 | 0.944534194 | 0.703998962 | 0.306752065 |
| C[C>T]A | 0.229175909 | 0.660531227 | 0.51053076 | 0.315403135 |
| C[G>A]A | 0.427622224 | 0.854784601 | 0.530812497 | 0.281118204 |
| C[G>C]A | 0.465135853 | 0.842972336 | 0.460175632 | 0.267170249 |
| C[G>T]A | 0.474905714 | 0.785785753 | 0.737680884 | 0.402586249 |
| C[T>A]A | 0.454713203 | 0.827839353 | 0.804482409 | 0.167939757 |
| C[T>C]A | 0.501687674 | 0.690666049 | 0.5711392 | 0.191191482 |
| C[T>G]A | 2.22E-16 | 1.100870417 | 2.22E-16 | 2.22E-16 |
| G[A>C]A | 0.320207587 | 0.628243161 | 0.575994492 | 0.255595461 |
| G[A>G]A | 0.337839474 | 0.60988516 | 0.504034206 | 0.209531023 |
| G[A>T]A | 0.38755997 | 0.610657645 | 0.511860088 | 0.128926645 |
| G[C>A]A | 0.460646428 | 0.759657462 | 0.702599837 | 0.194502796 |
| G[C>G]A | 0.344026027 | 0.602056838 | 0.554084566 | 0.287847639 |
| G[C>T]A | 0.524714874 | 0.889477498 | 0.561812272 | 0.291995887 |
| G[G>A]A | 0.388533729 | 0.616422584 | 0.644150096 | 0.27371605 |
| G[G>C]A | 0.482932751 | 0.879937178 | 0.58713829 | 0.248589549 |
| G[G>T]A | 0.300659375 | 0.489710317 | 0.473468491 | 0.299482897 |
| G[T>A]A | 2.22E-16 | 0.089866973 | 2.22E-16 | 2.22E-16 |
| G[T>C]A | 0.351899084 | 0.76367171 | 0.565661476 | 0.324746317 |
| G[T>G]A | 0.491551759 | 0.404185965 | 0.369461243 | 2.22E-16 |
| T[A>G]A | 1.048939984 | 2.22E-16 | 2.22E-16 | 0.262998647 |
| T[C>A]A | 2.22E-16 | 2.22E-16 | 0.128797987 | 2.22E-16 |
| T[C>G]A | 0.09535919 | 2.22E-16 | 0.347942514 | 2.22E-16 |
| T[C>T]A | 0.417967624 | 0.629252138 | 0.689462462 | 0.120860101 |
| T[G>A]A | 0.296277257 | 0.743877972 | 0.596260206 | 0.107214129 |
| T[T>C]A | 0.380142855 | 0.702872422 | 0.712986393 | 0.209267179 |
| T[T>G]A | 2.22E-16 | 2.22E-16 | 2.22E-16 | 0.080763139 |
| AA[C>A] | 0.541590436 | 0.695602774 | 0.567418758 | 0.405943506 |
| AA[G>A] | 0.435998932 | 0.612174799 | 0.523031751 | 0.220594985 |
| AA[T>A] | 0.73534427 | 0.985821812 | 0.78736213 | 0.37078295 |
| AC[C>A] | 0.464046509 | 0.572590939 | 0.527542787 | 0.220427618 |
| AC[G>A] | 0.426045973 | 0.741266151 | 0.65679764 | 0.409300386 |
| AC[T>A] | 0.328345947 | 0.936694616 | 0.481912513 | 0.20307325 |
| AG[C>A] | 0.423130066 | 0.722054345 | 0.428468596 | 0.23329302 |
| AG[G>A] | 0.43408512 | 0.642913543 | 0.712985421 | 0.411419939 |
| AG[T>A] | 2.22E-16 | 2.22E-16 | 1.037743783 | 2.22E-16 |
| AT[C>A] | 0.423969433 | 0.645865544 | 0.579175599 | 0.195532817 |
| AT[G>A] | 0.414569632 | 0.580025742 | 0.554252458 | 0.055853011 |
| AT[T>A] | 0.514771584 | 0.941927973 | 0.637859359 | 0.307372417 |
| CA[C>A] | 0.384313899 | 0.569543504 | 0.610246142 | 0.145270404 |
| CA[G>A] | 0.389959032 | 0.728649657 | 0.644465503 | 0.225356109 |
| CA[T>A] | 0.413062331 | 0.655414635 | 0.612151952 | 0.177988085 |
| CC[C>A] | 0.523057382 | 0.843677003 | 0.74538751 | 0.195875229 |
| CC[G>A] | 0.527540145 | 0.722898755 | 0.555572312 | 0.349678724 |
| CC[T>A] | 0.428062109 | 0.698209838 | 0.505977488 | 0.203886336 |
| CG[C>A] | 0.480885751 | 0.70092629 | 0.509143469 | 0.160743288 |
| CG[G>A] | 0.424815277 | 0.514734941 | 0.492553555 | 0.073709436 |
| CG[T>A] | 0.088298925 | 0.856544126 | 0.468512641 | 2.22E-16 |
| CT[C>A] | 0.507186943 | 0.640802768 | 0.360493883 | 0.239404392 |
| CT[G>A] | 0.458551945 | 0.76121069 | 0.617837014 | 0.387538046 |
| CT[T>A] | 0.403543502 | 0.676610759 | 0.3938665 | 0.207855805 |
| GA[C>A] | 0.334656125 | 0.512515343 | 0.59795175 | 0.322030813 |
| GA[G>A] | 0.377672202 | 0.803478936 | 0.58114797 | 0.463124742 |
| GA[T>A] | 0.224276052 | 0.661820004 | 0.251397273 | 0.089886104 |
| GC[C>A] | 0.441791791 | 0.743369181 | 0.596463485 | 0.143180137 |
| GC[G>A] | 0.497489545 | 0.729252914 | 0.529369713 | 0.185718922 |
| GC[T>A] | 0.474759107 | 0.850214818 | 0.626476813 | 0.233338142 |
| GG[C>A] | 0.417327962 | 0.759614916 | 0.483512482 | 0.229330677 |
| GG[G>A] | 0.443287185 | 0.789677895 | 0.582465715 | 0.293118923 |
| GG[T>A] | 0.45258864 | 0.723451474 | 0.357244745 | 0.231479674 |
| GT[C>A] | 0.2917353 | 0.625029719 | 0.441147921 | 0.318123025 |
| GT[G>A] | 0.485141205 | 0.97155975 | 0.768129966 | 0.327439923 |
| GT[T>A] | 0.418076333 | 0.685418124 | 0.799365498 | 0.210600055 |
| TA[C>A] | 0.283503293 | 0.273865305 | 0.292186449 | 2.22E-16 |
| TC[C>A] | 0.261969574 | 0.569015667 | 0.594852427 | 0.378620031 |
| TC[G>A] | 0.365940851 | 0.715546683 | 0.50655361 | 0.242460173 |
| TC[T>A] | 0.600896264 | 0.959198802 | 0.599084679 | 0.300284556 |
| TG[C>A] | 0.812225461 | 2.22E-16 | 2.22E-16 | 0.289338043 |
| TG[G>A] | 0.183322151 | 0.33949462 | 0.358996053 | 0.141072957 |
| TG[T>A] | 0.775626675 | 1.032248709 | 2.22E-16 | 2.22E-16 |
| TT[C>A] | 0.298551805 | 0.576031681 | 0.400709069 | 0.160872374 |
| TT[G>A] | 0.548609185 | 0.642046208 | 0.505288601 | 0.241842843 |
| TT[T>A] | 0.235712659 | 2.22E-16 | 0.421182396 | 2.22E-16 |
| [A>C]AC | 0.399426111 | 0.638914593 | 0.657525465 | 0.21837562 |
| [A>C]CC | 0.484162835 | 0.947624522 | 0.509734963 | 0.028052747 |
| [A>C]GC | 0.625951291 | 0.978621501 | 0.84939585 | 0.286512156 |
| [A>C]TC | 0.455678026 | 0.839825869 | 0.645540692 | 0.17426614 |
| [A>G]AC | 0.361554458 | 0.799541645 | 0.62680147 | 0.186659882 |
| [A>G]CC | 0.270929926 | 0.729854902 | 0.467146919 | 0.271698033 |
| [A>G]GC | 0.319060174 | 0.730950881 | 0.504971828 | 0.267018453 |
| [A>G]TC | 0.577817634 | 0.610851054 | 0.749884074 | 0.235678148 |
| [A>T]AC | 2.22E-16 | 0.758291205 | 0.611095351 | 2.22E-16 |
| [A>T]CC | 0.341971353 | 0.72479296 | 0.648484724 | 0.143071719 |
| [A>T]GC | 0.633987514 | 0.893358434 | 0.814026517 | 0.269345705 |
| [A>T]TC | 0.636216216 | 0.994379329 | 0.68357943 | 0.325076433 |
| [C>A]AC | 0.331693326 | 0.610085788 | 0.636512887 | 0.194509236 |
| [C>A]CC | 0.343237462 | 0.752754187 | 0.739055695 | 0.238499302 |
| [C>A]GC | 0.338267456 | 0.5109187 | 0.569944336 | 0.242147726 |
| [C>A]TC | 0.523046966 | 0.868309919 | 0.759689978 | 0.344288941 |
| [C>G]AC | 0.597354465 | 0.750480279 | 0.702855102 | 0.069972816 |
| [C>G]CC | 0.397042016 | 0.703052178 | 0.609083843 | 0.168560742 |
| [C>G]GC | 0.417336007 | 0.538702831 | 0.595463573 | 0.305494692 |
| [C>G]TC | 0.519414446 | 0.714123276 | 0.655908229 | 0.276276044 |
| [C>T]AC | 0.477126101 | 0.824001723 | 0.683625993 | 0.361785377 |
| [C>T]CC | 0.411318975 | 0.60515277 | 0.489021266 | 0.123527885 |
| [C>T]GC | 0.454831821 | 0.376029846 | 0.567687248 | 0.218952137 |
| [C>T]TC | 0.459838377 | 0.685325347 | 0.615797331 | 0.233293339 |
| [G>A]AC | 0.595784553 | 0.765410049 | 0.768751061 | 0.299947189 |
| [G>A]CC | 0.381033736 | 0.68209867 | 0.604358368 | 0.180997687 |
| [G>A]GC | 0.443115096 | 0.810057175 | 0.60213204 | 0.17737177 |
| [G>A]TC | 0.453344703 | 0.791186896 | 0.649807271 | 0.362690886 |
| [G>C]AC | 0.471142102 | 0.740035841 | 0.615683207 | 0.149164555 |
| [G>C]CC | 0.395343318 | 0.585688467 | 0.591655627 | 0.164244504 |
| [G>C]GC | 0.463169242 | 0.756033823 | 0.705675392 | 0.391184318 |
| [G>C]TC | 0.49785298 | 0.861624702 | 0.730179424 | 0.431773885 |
| [G>T]AC | 0.506877665 | 0.758936558 | 0.558047085 | 0.364711552 |
| [G>T]CC | 0.305642675 | 0.728915599 | 0.456434746 | 0.342645555 |
| [G>T]GC | 0.614486463 | 0.627736566 | 0.499341186 | 0.192959289 |
| [G>T]TC | 0.418713402 | 0.551116047 | 0.46871312 | 0.072626042 |
| [T>A]AC | 0.598581757 | 1.000910253 | 0.371680299 | 2.22E-16 |
| [T>A]CC | 0.540723485 | 0.890330839 | 0.683767781 | 0.269793194 |
| [T>A]GC | 0.350534906 | 0.621214402 | 0.587949756 | 0.352139586 |
| [T>A]TC | 0.442917535 | 0.515341405 | 0.446860818 | 0.155827294 |
| [T>C]AC | 0.530905276 | 0.736478984 | 0.615630381 | 0.25984342 |
| [T>C]CC | 0.418699565 | 0.648060524 | 0.526931109 | 0.31963326 |
| [T>C]GC | 0.559654821 | 0.988328217 | 0.760414976 | 0.279990675 |
| [T>C]TC | 0.370028578 | 0.661159896 | 0.44967077 | 0.196943609 |
| [T>G]AC | 0.307087186 | 0.59587742 | 0.779575377 | 0.187108733 |
| [T>G]CC | 0.311768379 | 0.866542419 | 0.676934536 | 0.1596812 |
| [T>G]GC | 0.361370555 | 0.6142552 | 0.565269082 | 0.096901107 |
| [T>G]TC | 0.585888499 | 0.960687779 | 0.885850026 | 0.365507796 |
| A[A>C]C | 0.442129519 | 0.6912708 | 0.691901074 | 0.236027503 |
| A[A>G]C | 0.492205936 | 0.68712163 | 0.603364227 | 0.121808363 |
| A[A>T]C | 0.472561688 | 0.537164147 | 0.598486377 | 0.101542266 |
| A[C>A]C | 0.276739843 | 0.568002146 | 0.627564673 | 0.215338054 |
| A[C>G]C | 0.270054081 | 0.744681862 | 0.392460335 | 0.128400208 |
| A[C>T]C | 0.447131226 | 0.732453756 | 0.568875435 | 0.2200939 |
| A[G>A]C | 0.362281809 | 0.569988849 | 0.518591508 | 0.051414404 |
| A[G>C]C | 0.304594513 | 0.64627634 | 0.703736447 | 0.193251377 |
| A[G>T]C | 0.528226981 | 0.775798901 | 0.622456959 | 0.180844365 |
| A[T>A]C | 0.542891825 | 0.979556352 | 0.71538807 | 0.374159743 |
| A[T>C]C | 0.338416168 | 0.768997787 | 0.642533144 | 0.340762 |
| A[T>G]C | 0.368310412 | 0.690469055 | 0.630459873 | 0.258158237 |
| C[A>C]C | 0.467318356 | 0.844395166 | 0.531747547 | 0.366172614 |
| C[A>G]C | 0.447334766 | 0.633158061 | 0.410037738 | 0.233991831 |
| C[A>T]C | 0.343619526 | 0.529345776 | 0.52918744 | 0.203061585 |
| C[C>A]C | 0.475870053 | 0.822302414 | 0.530793603 | 0.194712137 |
| C[C>G]C | 0.378644303 | 0.765089826 | 0.591068389 | 0.385275567 |
| C[C>T]C | 0.36506611 | 0.682370285 | 0.592814643 | 0.156572388 |
| C[G>A]C | 0.439917761 | 0.513213958 | 0.535956059 | 0.355253288 |
| C[G>C]C | 0.555932675 | 0.67331168 | 0.675623601 | 0.153773583 |
| C[G>T]C | 0.368043857 | 0.797921142 | 0.376142078 | 0.171401747 |
| C[T>A]C | 0.586335891 | 0.676399379 | 0.512296954 | 0.116396116 |
| C[T>C]C | 0.422725912 | 0.649202569 | 0.666011977 | 0.178064431 |
| C[T>G]C | 0.307833093 | 0.615466438 | 0.29199834 | 0.199617743 |
| G[A>C]C | 0.335525162 | 0.698552797 | 0.625375506 | 0.281740733 |
| G[A>G]C | 0.395968999 | 0.625126526 | 0.509379326 | 0.292289457 |
| G[A>T]C | 0.425146561 | 0.795525021 | 0.516393236 | 0.165524794 |
| G[C>A]C | 0.444388517 | 0.820374736 | 0.555913763 | 0.183577514 |
| G[C>G]C | 0.285288869 | 0.725585761 | 0.502194552 | 0.323108498 |
| G[C>T]C | 0.609994356 | 0.85591852 | 0.64604999 | 0.275499645 |
| G[G>A]C | 0.424764647 | 0.680894438 | 0.464134075 | 0.21833497 |
| G[G>C]C | 0.433837488 | 0.814273079 | 0.559271527 | 0.160200745 |
| G[G>T]C | 0.20850287 | 0.511789169 | 0.601583605 | 0.207232105 |
| G[T>A]C | 0.483960156 | 0.941762183 | 0.460049828 | 0.417507014 |
| G[T>C]C | 0.437071211 | 0.911382566 | 0.652977748 | 0.321530152 |
| G[T>G]C | 0.337679067 | 0.814967113 | 0.637400671 | 0.077445482 |
| T[A>C]C | 0.423634816 | 0.624164323 | 0.550548672 | 2.22E-16 |
| T[A>G]C | 0.400116699 | 0.66217768 | 0.715373636 | 0.289257568 |
| T[A>T]C | 0.525815417 | 0.795575338 | 0.728448893 | 0.284984779 |
| T[C>A]C | 0.463080605 | 0.624643672 | 0.517757405 | 0.178244482 |
| T[C>G]C | 0.563723301 | 0.677128199 | 0.543771998 | 0.171056288 |
| T[C>T]C | 0.446271246 | 0.840537367 | 0.590405357 | 0.116196217 |
| T[G>A]C | 0.400524152 | 0.567031161 | 0.484016478 | 0.113132252 |
| T[G>C]C | 0.390336291 | 0.599953604 | 0.541445399 | 0.248390254 |
| T[G>T]C | 0.53539542 | 0.817664656 | 0.532448116 | 0.305669107 |
| T[T>A]C | 0.48847004 | 0.915548617 | 0.647184394 | 0.370510527 |
| T[T>C]C | 0.374465045 | 0.696628213 | 0.541938434 | 0.253504411 |
| T[T>G]C | 0.529019882 | 0.684476422 | 0.431046928 | 0.15734415 |
| AA[A>C] | 0.535389622 | 0.883617932 | 0.687462247 | 0.261647702 |
| AA[G>C] | 0.418745933 | 0.72986324 | 0.609819471 | 0.201549156 |
| AA[T>C] | 0.657065014 | 0.697500616 | 0.570251313 | 0.337378406 |
| AC[A>C] | 0.410277952 | 0.725743696 | 0.723884094 | 0.123865409 |
| AC[G>C] | 0.40851282 | 0.750543863 | 0.571636023 | 0.283317195 |
| AC[T>C] | 0.519980967 | 0.878848275 | 0.565355679 | 0.219800396 |
| AG[A>C] | 0.450219154 | 0.733466104 | 0.513759165 | 0.26622266 |
| AG[G>C] | 0.362678869 | 0.525583023 | 0.358392835 | 0.130348511 |
| AG[T>C] | 0.442604221 | 0.770827514 | 0.563381961 | 0.362057409 |
| AT[A>C] | 0.242824968 | 0.777823721 | 0.585407484 | 0.219038138 |
| AT[G>C] | 0.190884611 | 0.690883046 | 0.255421493 | 0.251570757 |
| AT[T>C] | 0.547502219 | 0.802737579 | 0.670352575 | 0.23191628 |
| CA[A>C] | 0.458590212 | 0.842500791 | 0.646597924 | 0.28586697 |
| CA[G>C] | 0.571632332 | 0.662160221 | 0.457338412 | 0.333571394 |
| CA[T>C] | 0.386295242 | 0.769711445 | 0.538919238 | 0.248804236 |
| CC[A>C] | 0.689712548 | 0.915497878 | 0.614207589 | 0.313991992 |
| CC[G>C] | 0.368083832 | 0.675805445 | 0.542882758 | 0.241775486 |
| CC[T>C] | 0.463274956 | 0.645215443 | 0.479049892 | 0.164255753 |
| CG[A>C] | 0.326935191 | 0.53889998 | 0.317537901 | 0.320496098 |
| CG[G>C] | 0.329123618 | 0.702584076 | 0.533618212 | 0.341932001 |
| CG[T>C] | 0.507842061 | 0.738919719 | 0.662624704 | 0.328060451 |
| CT[A>C] | 0.346951549 | 0.607021211 | 0.297744589 | 0.242913577 |
| CT[G>C] | 0.303351798 | 0.789539399 | 0.657707202 | 0.230100969 |
| CT[T>C] | 0.365414453 | 0.551153153 | 0.596227224 | 0.36419546 |
| GA[A>C] | 0.236052096 | 0.613762372 | 0.52389672 | 0.206778124 |
| GA[G>C] | 0.383915893 | 0.718599973 | 0.505985702 | 0.235611731 |
| GA[T>C] | 0.622217965 | 1.083648087 | 0.596725641 | 0.010583143 |
| GC[A>C] | 0.339289849 | 0.661244746 | 0.549767363 | 0.232224175 |
| GC[G>C] | 0.324130819 | 0.859781291 | 0.738570562 | 0.145649269 |
| GC[T>C] | 0.39184536 | 0.704379101 | 0.534716908 | 0.216322311 |
| GG[A>C] | 0.265186062 | 0.904828875 | 0.497769358 | 0.272356096 |
| GG[G>C] | 0.354069835 | 0.77509415 | 0.714333972 | 0.198889697 |
| GG[T>C] | 0.513852948 | 0.777304535 | 0.650239782 | 0.29362057 |
| GT[A>C] | 0.321253887 | 0.760349734 | 0.437381522 | 0.247320526 |
| GT[G>C] | 0.556498703 | 0.698367349 | 0.523857599 | 0.213355311 |
| GT[T>C] | 0.491563071 | 0.770005409 | 0.644165172 | 0.281148095 |
| TA[T>C] | 0.311312871 | 0.42041657 | 0.535405085 | 0.133032324 |
| TC[A>C] | 0.38366108 | 0.612234483 | 0.629461087 | 0.276874925 |
| TC[G>C] | 0.449354655 | 0.717922154 | 0.654466993 | 0.223713115 |
| TC[T>C] | 0.319417623 | 0.522037821 | 0.554281115 | 0.334887336 |
| TG[G>C] | 0.255677774 | 0.698219921 | 0.43686748 | 0.403724825 |
| TG[T>C] | 0.378726757 | 0.66566065 | 0.475002457 | 0.159201663 |
| TT[A>C] | 0.341671744 | 0.556992963 | 0.420903759 | 0.271383654 |
| TT[G>C] | 0.49426571 | 0.589233176 | 0.632830378 | 0.330565395 |
| TT[T>C] | 0.465809344 | 0.64421093 | 0.610284496 | 0.330267686 |
| [A>C]AG | 0.396899538 | 0.593387993 | 0.601567027 | 0.170631292 |
| [A>C]CG | 0.565888327 | 1.071688874 | 0.755630374 | 0.441015351 |
| [A>C]GG | 0.517659875 | 0.628595702 | 0.740708801 | 0.173663118 |
| [A>C]TG | 0.402626077 | 0.675564233 | 0.607228261 | 0.381468891 |
| [A>G]AG | 0.3118764 | 0.766388947 | 0.550558525 | 0.20693992 |
| [A>G]CG | 0.295206421 | 0.777756279 | 0.62228522 | 0.293280198 |
| [A>G]GG | 0.341746585 | 0.498243143 | 0.460310452 | 0.190339885 |
| [A>G]TG | 0.496301151 | 0.711103889 | 0.471620557 | 0.304982216 |
| [A>T]AG | 0.046632859 | 2.22E-16 | 2.22E-16 | 2.22E-16 |
| [A>T]CG | 0.387827591 | 0.740593314 | 0.523438237 | 0.216518495 |
| [A>T]GG | 0.162425582 | 0.548607898 | 0.693920279 | 0.521192751 |
| [A>T]TG | 0.401281811 | 0.778668994 | 0.607066569 | 0.250607587 |
| [C>A]AG | 0.529390448 | 0.954839241 | 0.765621203 | 0.269940685 |
| [C>A]CG | 2.22E-16 | 0.168175784 | 0.22863296 | 0.252712542 |
| [C>A]GG | 0.422865304 | 0.797100037 | 0.638043491 | 0.207321087 |
| [C>A]TG | 0.552283347 | 0.648767392 | 0.542810047 | 0.33962519 |
| [C>G]AG | 0.363835293 | 0.577283724 | 0.411571457 | 0.173820916 |
| [C>G]CG | 0.335313127 | 0.761180681 | 0.695928538 | 0.197458954 |
| [C>G]GG | 0.534519249 | 0.622795202 | 0.534873276 | 0.184101194 |
| [C>G]TG | 0.47258497 | 0.838567131 | 0.633770758 | 0.2623708 |
| [C>T]AG | 0.370794554 | 0.582673321 | 0.507948398 | 0.115856761 |
| [C>T]CG | 0.474473394 | 0.672678887 | 0.848619954 | 2.22E-16 |
| [C>T]GG | 0.408902033 | 0.682146485 | 0.58724045 | 0.161805682 |
| [C>T]TG | 0.457077926 | 0.901247804 | 0.616156802 | 0.413235751 |
| [G>A]AG | 0.469833408 | 0.697244534 | 0.668355527 | 0.196070887 |
| [G>A]CG | 0.388763501 | 0.522700902 | 0.835268942 | 0.271626863 |
| [G>A]GG | 0.419352643 | 0.731314593 | 0.518414321 | 0.336130409 |
| [G>A]TG | 0.220687827 | 0.726163887 | 0.652469547 | 0.049642874 |
| [G>C]AG | 0.471743118 | 0.631266931 | 0.653233512 | 0.353682955 |
| [G>C]CG | 0.611983347 | 0.927926631 | 0.765451192 | 0.309569744 |
| [G>C]GG | 0.330149148 | 0.661377819 | 0.606850924 | 0.209189054 |
| [G>C]TG | 0.378177225 | 0.628713168 | 0.431808644 | 0.161307423 |
| [G>T]AG | 0.385994394 | 0.486239928 | 0.4945652 | 0.254788676 |
| [G>T]CG | 0.493760384 | 0.984486012 | 0.793255484 | 0.397086251 |
| [G>T]GG | 0.275279107 | 0.731166234 | 0.646575367 | 2.22E-16 |
| [G>T]TG | 0.511854177 | 0.741717172 | 0.609527049 | 0.294934682 |
| [T>A]CG | 0.453332048 | 0.688392226 | 0.854082868 | 0.405317635 |
| [T>A]GG | 2.22E-16 | 0.532660493 | 0.378102832 | 2.22E-16 |
| [T>A]TG | 0.468768382 | 0.651048716 | 0.528368849 | 0.304803115 |
| [T>C]AG | 0.354409731 | 2.22E-16 | 2.22E-16 | 2.22E-16 |
| [T>C]CG | 0.391282067 | 0.632429891 | 0.476782839 | 0.236632199 |
| [T>C]GG | 0.346095949 | 0.620888827 | 0.390590986 | 0.483180501 |
| [T>C]TG | 0.403300556 | 0.725092997 | 0.573180073 | 0.119754308 |
| [T>G]AG | 0.046632859 | 2.22E-16 | 2.22E-16 | 2.22E-16 |
| [T>G]CG | 0.491764831 | 0.606797108 | 0.547577395 | 0.177890028 |
| [T>G]GG | 0.579742043 | 0.667268799 | 0.570738227 | 0.100012413 |
| [T>G]TG | 0.273475986 | 0.596888823 | 0.486361697 | 0.258492142 |
| A[A>C]G | 0.403116565 | 0.579302801 | 0.626844244 | 0.135992176 |
| A[A>G]G | 0.678481818 | 0.829156062 | 0.733481982 | 0.217519375 |
| A[A>T]G | 0.552987933 | 0.944575402 | 0.750656535 | 0.327540715 |
| A[C>A]G | 0.530304424 | 0.620443323 | 0.636861778 | 0.208811904 |
| A[C>G]G | 0.483342464 | 0.802942483 | 0.517522205 | 0.368030054 |
| A[C>T]G | 0.369100398 | 0.710305452 | 0.593940664 | 0.22802152 |
| A[G>A]G | 0.538629371 | 0.822523533 | 0.729977353 | 0.056251069 |
| A[G>C]G | 0.476934688 | 0.51157262 | 0.505278832 | 0.26286899 |
| A[G>T]G | 0.568632837 | 0.724564904 | 0.616549853 | 0.325866683 |
| A[T>A]G | 0.412401772 | 0.584757718 | 0.466042182 | 0.131272658 |
| A[T>C]G | 0.472582316 | 0.729931215 | 0.655772863 | 0.051316015 |
| A[T>G]G | 0.464074884 | 0.768452749 | 0.610002538 | 0.271862732 |
| C[A>C]G | 0.399588443 | 0.585997257 | 0.561434737 | 0.176993013 |
| C[A>G]G | 0.384826588 | 0.700139464 | 0.527926538 | 0.16544235 |
| C[A>T]G | 0.5037645 | 0.657662806 | 0.689839787 | 0.227002791 |
| C[C>A]G | 0.412948528 | 0.560809938 | 0.540406686 | 0.1197253 |
| C[C>G]G | 0.390960382 | 0.515374957 | 0.503813851 | 0.221699142 |
| C[C>T]G | 0.443788636 | 0.671514671 | 0.594260383 | 0.245523228 |
| C[G>A]G | 0.354404703 | 0.718560393 | 0.510134021 | 0.276270473 |
| C[G>C]G | 0.358514954 | 0.868311498 | 0.535367171 | 0.080047311 |
| C[G>T]G | 0.516264018 | 0.978603671 | 0.545656579 | 0.320354758 |
| C[T>A]G | 0.334291624 | 0.664771969 | 0.637986252 | 0.059850929 |
| C[T>C]G | 0.373450221 | 0.802067844 | 0.743582071 | 0.191021534 |
| C[T>G]G | 0.484987886 | 0.747453901 | 0.549757292 | 0.370529801 |
| G[A>C]G | 0.471867217 | 0.840713292 | 0.594416027 | 0.302599152 |
| G[A>G]G | 0.328205007 | 0.646844301 | 0.698705635 | 0.165830986 |
| G[A>T]G | 0.387506419 | 0.521650218 | 0.461652649 | 0.186143788 |
| G[C>A]G | 0.48174836 | 0.851716867 | 0.585917838 | 0.047219843 |
| G[C>G]G | 0.472901851 | 0.694547787 | 0.702552107 | 0.304841998 |
| G[C>T]G | 0.450613396 | 0.655900137 | 0.510201973 | 0.170824326 |
| G[G>A]G | 0.319972085 | 0.631203908 | 0.684728629 | 0.354445431 |
| G[G>C]G | 0.414231766 | 0.716046879 | 0.444432037 | 0.310594561 |
| G[G>T]G | 0.460508574 | 0.731891902 | 0.535328521 | 0.266867777 |
| G[T>A]G | 0.34069142 | 0.85569168 | 0.780369242 | 0.261344463 |
| G[T>C]G | 0.511296547 | 0.638055972 | 0.717120163 | 0.110287508 |
| G[T>G]G | 0.512162352 | 0.78645748 | 0.58365887 | 0.34216775 |
| T[A>G]G | 2.22E-16 | 2.22E-16 | 2.22E-16 | 0.484578831 |
| T[C>A]G | 0.493492384 | 0.264601494 | 0.491309569 | 2.22E-16 |
| T[C>G]G | 0.224223934 | 0.615664102 | 0.4770915 | 0.319153897 |
| T[C>T]G | 0.321061602 | 0.575775128 | 0.529149738 | 0.187922712 |
| T[G>A]G | 0.563921298 | 0.844843774 | 0.550986131 | 0.151189562 |
| T[G>C]G | 0.501762454 | 0.980812585 | 0.680810121 | 0.346947924 |
| T[G>T]G | 0.249116391 | 0.670203815 | 0.564165202 | 0.349587175 |
| T[T>A]G | 0.697210935 | 0.645405525 | 0.845694935 | 0.180218433 |
| T[T>C]G | 0.393399696 | 0.776712078 | 0.649143559 | 0.373554662 |
| T[T>G]G | 0.338095334 | 0.602370305 | 0.51792497 | 0.176757695 |
| AA[A>G] | 0.616605237 | 1.014113291 | 0.771307771 | 0.250225059 |
| AA[C>G] | 0.281047455 | 0.70716245 | 0.754273438 | 0.168853724 |
| AA[T>G] | 0.626566093 | 0.707816631 | 0.671986926 | 2.22E-16 |
| AC[A>G] | 0.412537134 | 0.469887499 | 0.550357924 | 0.31644992 |
| AC[C>G] | 0.407693412 | 0.811682003 | 0.523535223 | 0.270860389 |
| AC[T>G] | 0.358582277 | 0.547017825 | 0.312119325 | 0.203535442 |
| AG[A>G] | 0.26938897 | 0.538668411 | 0.34660954 | 0.290777464 |
| AG[C>G] | 0.618813788 | 0.771545709 | 0.69936791 | 0.251999038 |
| AG[T>G] | 0.428322661 | 0.814441336 | 0.662416231 | 0.247433722 |
| AT[A>G] | 0.488975282 | 0.55998504 | 0.469613421 | 0.21699263 |
| AT[C>G] | 0.299157242 | 0.695212955 | 0.437809306 | 0.195544317 |
| AT[T>G] | 0.566250876 | 0.834073456 | 0.521454424 | 0.383263719 |
| CA[A>G] | 0.355805632 | 0.792485547 | 0.781789648 | 0.230802607 |
| CA[C>G] | 0.338381999 | 0.810265089 | 0.587356335 | 0.33465238 |
| CA[T>G] | 0.430276103 | 0.684028632 | 0.490666867 | 0.325390198 |
| CC[A>G] | 0.508541987 | 0.59711348 | 0.504601637 | 0.115972616 |
| CC[C>G] | 0.367585329 | 0.632525514 | 0.47646864 | 0.204122869 |
| CC[T>G] | 0.355636917 | 0.535329881 | 0.40065655 | 0.264937159 |
| CG[A>G] | 0.479666557 | 0.575167714 | 0.791738097 | 0.48768911 |
| CG[C>G] | 0.448307958 | 0.533415964 | 0.34629641 | 0.130793326 |
| CG[T>G] | 0.403365179 | 0.659256342 | 0.711652165 | 0.048887983 |
| CT[A>G] | 0.498302818 | 0.681856063 | 0.744913535 | 0.348255951 |
| CT[C>G] | 0.489947347 | 0.783642464 | 0.622610253 | 0.204232444 |
| CT[T>G] | 0.367192768 | 0.58705129 | 0.707851551 | 0.259460299 |
| GA[A>G] | 0.390416391 | 0.88056437 | 0.605542324 | 0.35585709 |
| GA[C>G] | 0.4969184 | 0.90600996 | 0.550490909 | 0.144466988 |
| GA[T>G] | 0.348740849 | 0.623364002 | 0.593718686 | 0.362626931 |
| GC[A>G] | 0.366328231 | 0.659885932 | 0.725074194 | 0.203010523 |
| GC[C>G] | 0.347398953 | 0.49762558 | 0.381705241 | 0.199996135 |
| GC[T>G] | 0.35297143 | 0.593704489 | 0.419453565 | 0.263919713 |
| GG[A>G] | 0.399975702 | 0.804398292 | 0.619882654 | 0.290392274 |
| GG[C>G] | 0.239978674 | 0.667242092 | 0.527675894 | 0.190749163 |
| GG[T>G] | 0.414430478 | 0.657500828 | 0.698553318 | 0.214974077 |
| GT[A>G] | 0.354019811 | 0.505416016 | 0.698666853 | 0.109305866 |
| GT[C>G] | 0.346702892 | 0.576099321 | 0.740138205 | 0.259247133 |
| GT[T>G] | 0.450076498 | 0.77651426 | 0.814816048 | 0.0916979 |
| TA[A>G] | 0.591035793 | 0.931615861 | 0.849037297 | 0.32094264 |
| TA[T>G] | 0.604331617 | 0.989850859 | 0.859470897 | 2.22E-16 |
| TC[A>G] | 0.479593066 | 0.6519563 | 0.585078443 | 0.104986942 |
| TC[C>G] | 0.466419749 | 0.809132175 | 0.558951913 | 0.317186924 |
| TC[T>G] | 0.507398557 | 0.708898407 | 0.606712502 | 0.306927427 |
| TG[C>G] | 0.123016092 | 0.461987125 | 0.29528362 | 2.22E-16 |
| TG[T>G] | 0.534658738 | 0.52437798 | 0.626809216 | 0.309388815 |
| TT[A>G] | 0.351282831 | 0.510928699 | 0.434607379 | 0.131428839 |
| TT[C>G] | 0.471224942 | 0.733648889 | 0.599906871 | 2.22E-16 |
| TT[T>G] | 0.378174808 | 0.596160441 | 0.539403925 | 0.122056545 |
| [A>C]AT | 0.477869606 | 0.633417784 | 0.586958015 | 0.475385861 |
| [A>C]CT | 0.318167299 | 0.473985276 | 0.470560969 | 0.169224854 |
| [A>C]GT | 0.633197634 | 0.91622977 | 0.68737282 | 0.326526276 |
| [A>C]TT | 0.55455622 | 1.102348106 | 0.839080062 | 0.409237248 |
| [A>G]AT | 0.450240421 | 0.578552195 | 0.566947659 | 0.148653792 |
| [A>G]CT | 0.42037385 | 0.883719898 | 0.547636184 | 0.266765501 |
| [A>G]GT | 0.381825273 | 0.680495906 | 0.69059316 | 0.274277816 |
| [A>G]TT | 0.486912578 | 0.657174211 | 0.479193107 | 0.212674507 |
| [A>T]AT | 0.558328194 | 0.794049778 | 0.737935258 | 0.258184846 |
| [A>T]CT | 0.378516308 | 0.658229763 | 0.556868874 | 0.287222153 |
| [A>T]GT | 0.468430469 | 0.599675576 | 0.448453115 | 0.394002213 |
| [A>T]TT | 0.46472352 | 0.629216719 | 0.667325034 | 0.225842281 |
| [C>A]AT | 0.568839418 | 0.687383282 | 0.539656852 | 0.218274648 |
| [C>A]CT | 0.387135999 | 0.634103215 | 0.606663947 | 0.205521237 |
| [C>A]GT | 0.373341813 | 0.784650502 | 0.43662584 | 0.191732813 |
| [C>A]TT | 0.480232539 | 0.80138586 | 0.596574559 | 0.249428914 |
| [C>G]AT | 0.515448585 | 0.614271483 | 0.601033019 | 0.151498534 |
| [C>G]CT | 0.460502851 | 0.693054344 | 0.709522971 | 0.242448602 |
| [C>G]GT | 0.41496546 | 0.777294723 | 0.527577579 | 0.081273819 |
| [C>G]TT | 0.426635454 | 0.788504718 | 0.501840672 | 0.308736879 |
| [C>T]AT | 0.346826529 | 0.603970358 | 0.543070847 | 0.205297786 |
| [C>T]CT | 0.348873734 | 0.726663893 | 0.682818478 | 0.259022456 |
| [C>T]GT | 0.41816212 | 0.694988949 | 0.650318517 | 0.145409147 |
| [C>T]TT | 0.378565888 | 0.814130868 | 0.55006277 | 0.338567801 |
| [G>A]AT | 0.338367907 | 0.8136516 | 0.651458822 | 0.333257903 |
| [G>A]CT | 0.259785111 | 0.634334998 | 0.581561138 | 0.300394042 |
| [G>A]GT | 0.403828996 | 0.681280312 | 0.552676848 | 0.126935221 |
| [G>A]TT | 0.384431443 | 0.810412503 | 0.55622198 | 0.213073931 |
| [G>C]AT | 0.437367211 | 0.619737553 | 0.548358904 | 0.229014335 |
| [G>C]CT | 0.498129399 | 0.826411636 | 0.593074012 | 0.350971335 |
| [G>C]GT | 0.537723967 | 0.987616608 | 0.686188238 | 0.396416959 |
| [G>C]TT | 0.315068557 | 0.693416918 | 0.525940098 | 0.27726574 |
| [G>T]AT | 0.324707148 | 0.783027166 | 0.446004934 | 0.113506388 |
| [G>T]CT | 0.40493681 | 0.864064133 | 0.611470283 | 0.16349479 |
| [G>T]GT | 0.423869869 | 0.910113833 | 0.821017053 | 0.185292194 |
| [G>T]TT | 0.407820881 | 0.573326874 | 0.605703667 | 0.135981026 |
| [T>A]AT | 0.505873824 | 0.605765952 | 0.558165016 | 0.18628443 |
| [T>A]CT | 0.553007387 | 1.041634028 | 0.849336604 | 0.374988612 |
| [T>A]GT | 0.504233658 | 0.815981772 | 0.595417145 | 0.300064893 |
| [T>A]TT | 0.538384618 | 0.556817591 | 0.632054221 | 0.275298871 |
| [T>C]AT | 0.541002673 | 0.797880289 | 0.497106623 | 0.202748285 |
| [T>C]CT | 0.267299566 | 0.677353371 | 0.583707795 | 0.21194872 |
| [T>C]GT | 0.518361909 | 0.605645659 | 0.566586786 | 0.075764979 |
| [T>C]TT | 0.39145072 | 0.75849276 | 0.530896508 | 0.295257699 |
| [T>G]AT | 0.379690057 | 0.863418421 | 0.516311068 | 0.175167255 |
| [T>G]CT | 0.502403769 | 0.754879057 | 0.580637766 | 0.228842497 |
| [T>G]GT | 0.318048022 | 0.638507027 | 0.465502568 | 0.137312218 |
| [T>G]TT | 0.374012785 | 0.820412498 | 0.660581048 | 0.280722089 |
| A[A>C]T | 0.390054375 | 0.506256849 | 0.50427612 | 0.225094651 |
| A[A>G]T | 0.434331317 | 0.73463812 | 0.465027626 | 0.169332285 |
| A[A>T]T | 0.372120527 | 0.814328545 | 0.762495287 | 0.399965785 |
| A[C>A]T | 0.398199671 | 0.478827551 | 0.742182234 | 0.266923578 |
| A[C>G]T | 0.374712831 | 0.704655146 | 0.705246225 | 0.33885673 |
| A[C>T]T | 0.301539946 | 0.606406262 | 0.614814354 | 0.131789418 |
| A[G>A]T | 0.476940305 | 0.735644794 | 0.52658036 | 0.090542217 |
| A[G>C]T | 0.424660205 | 0.75586687 | 0.620666225 | 0.252528183 |
| A[G>T]T | 0.482587016 | 0.558484914 | 0.64111513 | 0.223636566 |
| A[T>A]T | 0.56719288 | 0.663998056 | 0.546757532 | 0.262194006 |
| A[T>C]T | 0.410357512 | 0.836109551 | 0.586878861 | 0.340385375 |
| A[T>G]T | 0.449466707 | 0.754265133 | 0.584303827 | 0.292824711 |
| C[A>C]T | 0.563854445 | 1.00361432 | 0.539135323 | 0.261116331 |
| C[A>G]T | 0.626529693 | 0.654670229 | 0.864705336 | 0.327842709 |
| C[A>T]T | 0.336740934 | 0.774079099 | 0.619822555 | 0.22314524 |
| C[C>A]T | 0.583000287 | 0.56451576 | 0.706110838 | 0.271279205 |
| C[C>G]T | 0.52191398 | 0.826187592 | 0.555611455 | 0.310563819 |
| C[C>T]T | 0.399769123 | 0.68864866 | 0.580112399 | 0.240027266 |
| C[G>A]T | 0.308165954 | 0.80722202 | 0.683689077 | 0.227892631 |
| C[G>C]T | 0.531547183 | 0.590986288 | 0.66198769 | 0.210564328 |
| C[G>T]T | 0.411282941 | 0.783076261 | 0.698541317 | 0.11275937 |
| C[T>A]T | 0.594869481 | 0.642338635 | 0.648118114 | 0.334654656 |
| C[T>C]T | 0.420740182 | 0.835720852 | 0.745981495 | 0.300479626 |
| C[T>G]T | 0.553531731 | 0.690625683 | 0.425107599 | 0.309649322 |
| G[A>C]T | 0.385301473 | 0.709210282 | 0.582237468 | 0.288757879 |
| G[A>G]T | 0.317071269 | 0.787296464 | 0.491837841 | 0.307681101 |
| G[A>T]T | 0.584837832 | 0.882071329 | 0.671924282 | 2.22E-16 |
| G[C>A]T | 0.396652934 | 0.659999793 | 0.727823489 | 0.195713082 |
| G[C>G]T | 0.461031175 | 0.716800706 | 0.636530104 | 0.362748922 |
| G[C>T]T | 0.311906018 | 0.665021712 | 0.614227865 | 0.337254344 |
| G[G>A]T | 0.361508877 | 0.663176825 | 0.62600736 | 0.148445581 |
| G[G>C]T | 0.346646006 | 0.572884458 | 0.589353943 | 0.282804266 |
| G[G>T]T | 0.544771363 | 0.756261107 | 0.736378479 | 0.164234186 |
| G[T>A]T | 0.498684538 | 0.974833539 | 0.706975538 | 0.351015702 |
| G[T>C]T | 0.492563387 | 0.764957855 | 0.594355002 | 2.22E-16 |
| G[T>G]T | 0.606031447 | 0.910697039 | 0.67577602 | 0.35617654 |
| T[A>C]T | 0.507409781 | 0.67015125 | 0.650162795 | 0.21974201 |
| T[A>G]T | 0.4157114 | 0.833672699 | 0.650504709 | 0.209069121 |
| T[A>T]T | 0.592401827 | 0.916279587 | 0.819090093 | 0.368921983 |
| T[C>A]T | 0.372706407 | 0.489641145 | 0.441271945 | 0.200728755 |
| T[C>G]T | 0.399809743 | 0.590852992 | 0.505404233 | 0.287182912 |
| T[C>T]T | 0.343153203 | 0.799933095 | 0.625671114 | 0.247278699 |
| T[G>A]T | 0.36286349 | 0.702396304 | 0.484930998 | 0.202308905 |
| T[G>C]T | 0.500927079 | 0.660400951 | 0.404596313 | 0.090993323 |
| T[G>T]T | 0.604236618 | 0.900185841 | 0.727745486 | 0.332347054 |
| T[T>A]T | 0.420706476 | 0.732400544 | 0.65584817 | 0.217043116 |
| T[T>C]T | 0.469843163 | 0.659102231 | 0.601178709 | 0.189233368 |
| T[T>G]T | 0.270255099 | 0.486185428 | 0.517624207 | 0.125537379 |
| AA[A>T] | 0.363465537 | 0.498461957 | 0.444732794 | 0.129906875 |
| AA[C>T] | 0.44855995 | 0.606440761 | 0.572278604 | 0.279244844 |
| AA[G>T] | 0.400064309 | 0.719905141 | 0.612467902 | 0.367608039 |
| AC[A>T] | 0.313184101 | 0.593339003 | 0.583705759 | 0.278748694 |
| AC[C>T] | 0.508877821 | 0.826186594 | 0.725607473 | 0.397218583 |
| AC[G>T] | 0.3548655 | 0.589953323 | 0.386652646 | 0.212231035 |
| AG[A>T] | 0.353398497 | 0.709868866 | 0.527505259 | 0.279646781 |
| AG[C>T] | 0.463634885 | 0.69656342 | 0.695380599 | 0.303567349 |
| AG[G>T] | 0.433825799 | 0.674981741 | 0.487607988 | 0.168559029 |
| AT[A>T] | 0.606030685 | 0.96434542 | 0.751583769 | 0.285963198 |
| AT[C>T] | 0.309233259 | 0.711695887 | 0.626937204 | 0.371621691 |
| AT[G>T] | 0.483717874 | 0.576619958 | 0.402021132 | 0.401378059 |
| CA[A>T] | 0.44180332 | 0.774123973 | 0.70800594 | 0.210990996 |
| CA[C>T] | 0.407996852 | 0.783226139 | 0.625707172 | 0.059522174 |
| CA[G>T] | 0.502677961 | 0.654467173 | 0.430201613 | 0.165708528 |
| CC[A>T] | 0.503232796 | 0.725282954 | 0.544484044 | 0.416271935 |
| CC[C>T] | 0.435843411 | 0.704849793 | 0.501421232 | 0.207176119 |
| CC[G>T] | 0.457355443 | 0.722117868 | 0.695002617 | 0.217080885 |
| CG[A>T] | 0.727829128 | 1.033594104 | 0.836948679 | 0.346919625 |
| CG[C>T] | 0.320364028 | 0.476701216 | 0.665421537 | 0.178141627 |
| CG[G>T] | 0.466562815 | 0.709188231 | 0.446046192 | 0.202369452 |
| CT[A>T] | 0.508614206 | 0.742555355 | 0.472067284 | 0.371590077 |
| CT[C>T] | 0.515939125 | 0.784754109 | 0.61089432 | 0.030507831 |
| CT[G>T] | 0.333785023 | 0.703379617 | 0.477677577 | 0.296978322 |
| GA[A>T] | 0.445517415 | 0.7677826 | 0.446237478 | 0.160090785 |
| GA[C>T] | 0.631036939 | 1.034037446 | 0.744594709 | 0.253732472 |
| GA[G>T] | 0.438064179 | 0.861404169 | 0.519549109 | 0.348594284 |
| GC[A>T] | 0.303307814 | 0.604148459 | 0.575072226 | 0.163130941 |
| GC[C>T] | 0.323138089 | 0.717685341 | 0.527128881 | 0.279598885 |
| GC[G>T] | 0.301450525 | 0.78273076 | 0.659523486 | 0.372304444 |
| GG[A>T] | 0.390337376 | 0.68587239 | 0.604281482 | 0.061913856 |
| GG[C>T] | 0.603905383 | 0.677602876 | 0.547129461 | 0.3206592 |
| GG[G>T] | 0.397008777 | 0.613470752 | 0.531399639 | 0.174660965 |
| GT[A>T] | 0.436464332 | 0.820225053 | 0.506997395 | 0.158138142 |
| GT[C>T] | 0.340785965 | 0.700248957 | 0.443529401 | 0.285053002 |
| GT[G>T] | 0.408067953 | 0.516413718 | 0.636830606 | 0.194031836 |
| TA[C>T] | 0.440610657 | 0.719330576 | 0.668217447 | 0.165504255 |
| TC[A>T] | 0.455180616 | 0.566502028 | 0.459035291 | 0.270579239 |
| TC[C>T] | 0.488665909 | 0.713755397 | 0.697326608 | 0.280590634 |
| TC[G>T] | 0.650857238 | 0.515578084 | 0.573647847 | 0.378403336 |
| TG[C>T] | 0.324688382 | 0.717929149 | 0.495606795 | 0.222912806 |
| TG[G>T] | 0.540738889 | 0.998008854 | 0.743017473 | 0.432919929 |
| TT[A>T] | 2.22E-16 | 0.954653648 | 2.22E-16 | 0.202302422 |
| TT[C>T] | 0.40740052 | 0.813887103 | 0.65521832 | 0.284508565 |
| TT[G>T] | 0.49516695 | 0.738232984 | 0.731672939 | 0.296594133 |
